# Predictive Biomarkers and Novel Treatments for the Progressive Fibrosing Phenotype in Interstitial Lung Disease Associated with Connective Tissue Disease

**DOI:** 10.3390/biomedicines13061463

**Published:** 2025-06-13

**Authors:** Sang Wan Chung

**Affiliations:** Division of Rheumatology, Department of Internal Medicine, Kyung Hee University College of Medicine, Kyung Hee University Hospital, Seoul 02447, Republic of Korea; wanyworld83@gmail.com

**Keywords:** connective tissue diseases-interstitial lung diseases, progressive fibrosing-interstitial lung disease, phenotype, biomarkers

## Abstract

Progressive fibrosing interstitial lung disease (PF-ILD) is a significant complication of connective tissue diseases (CTDs), particularly in systemic sclerosis (SSc), rheumatoid arthritis (RA), and idiopathic inflammatory myopathies (IIM). Despite clinical similarities with idiopathic pulmonary fibrosis (IPF), CTD-associated ILDs exhibit distinct pathogenetic and immunologic features. **Objective:** This review aims to summarize key predictive biomarkers and current treatment strategies associated with the progressive fibrosing phenotype in SSc-ILD, RA-ILD, and IIM-ILD. **Methods:** We conducted a focused literature search of PubMed and Scopus databases covering publications from January 2010 to February 2024. Included studies evaluated serum, cellular, or genetic biomarkers with predictive value for disease progression or treatment response. Only peer-reviewed English-language articles were included. Exclusion criteria encompassed single case reports and editorials. **Results:** Several biomarkers, including KL-6, SP-D, CXCL4, and anti-MDA5, demonstrate potential in predicting fibrotic progression in CTD-ILDs. However, variability in sensitivity and specificity across CTD subtypes limits broad clinical applicability. Therapeutic agents such as nintedanib and pirfenidone show efficacy in slowing lung function decline. Biologics including rituximab and tocilizumab offer additional options, particularly in immunologically active diseases. **Conclusion:** Although promising biomarkers and therapies are emerging, no single marker or intervention currently predicts or modifies PF-ILD outcomes across all CTD subsets. Prospective studies and integrative biomarker panels are needed to improve patient stratification and guide therapy.

## 1. Introduction

Connective tissue diseases (CTDs) are a group of systemic rheumatological disorders characterized by autoimmune-mediated inflammation with variable presentations. CTDs include rheumatoid arthritis (RA), idiopathic inflammatory myopathies (IIM), systemic sclerosis (SSc), and systemic lupus erythematosus [1]. Various organs can be affected by CTD. Among them, the lungs are commonly affected by autoimmunity-mediated organ damage in patients with CTD. Pulmonary involvement is the leading cause of morbidity and mortality in CTD patients.

Among the pulmonary complications observed in rheumatic diseases, interstitial lung disease (ILD) stands out as the most prevalent and clinically significant. ILD comprises a broad spectrum of conditions that share overlapping clinical presentations, imaging patterns, and histopathological findings, all involving diffuse damage to lung parenchyma [2] While a variety of factors—including environmental toxins and medications—may cause ILD, connective tissue disease remains one of its principal origins. Earlier diagnostic frameworks classified ILD based on histopathological subtypes as per the 2013 ATS/ERS criteria, categorizing it into entities like usual interstitial pneumonia (UIP), nonspecific interstitial pneumonia (NSIP), and organizing pneumonia, each associated with distinct clinical behaviors [3]. However, such categorization has limitations when it comes to guiding treatment or predicting disease progression. A newer classification based on disease behavior—particularly the presence of progressive fibrosis—has been introduced to better reflect clinical realities. The progressive fibrosing phenotype (PF-ILD) is defined by a decline in respiratory function, ongoing fibrosis, and symptom exacerbation despite therapy [4]. This fibrosing process results from a multifaceted interaction of immune, genetic, and environmental factors, where immune cell activation and epithelial injury lead to excessive deposition of extracellular matrix proteins, largely mediated by cytokines like TGF-β, IL-6, and PDGF. Unraveling these mechanisms is crucial to identifying predictive biomarkers and refining phenotypic classification across CTD-ILD [2].

The variable presentation and course of CTD-ILD necessitate an in-depth understanding of the phenotype–endotype relation to predict disease outcomes and inform targeted therapies. The relation between the phenotype and endotype—the phenotypic expression of the disease and underlying molecular mechanisms—is critical for patient stratification. Biomarkers help bridge the gap between these concepts by enabling the identification of disease subtypes and the prediction of disease trajectories.

## 2. Methods

We conducted a focused literature review using PubMed, Scopus, and Web of Science databases from January 2014 to February 2025 (accessed on 19 May 2025). The search terms included “progressive fibrosing interstitial lung disease”, “connective tissue disease”, “biomarkers”, “RA-ILD”, “SSc-ILD”, “IIM-ILD”, and “antifibrotic therapy.” Boolean operators (AND, OR) were used to refine the search. Only articles published in English were included.

We included original studies, meta-analyses, and systematic reviews that evaluated biomarkers or therapeutic outcomes in PF-ILD associated with RA, SSc, or IIM. Studies were eligible if they assessed disease progression, response to antifibrotic therapy, or biomarker sensitivity/specificity. Excluded articles included case reports, editorials, and non-peer-reviewed publications.

After screening titles and abstracts, full texts of eligible studies were reviewed. Relevant data were extracted, including study design, population characteristics, type of biomarker evaluated, outcome measures (e.g., FVC decline, mortality), and key findings.

## 3. Progressive Fibrosing Phenotype in CTD-ILD

The progressive fibrosing (PF) phenotype in CTD-ILD is characterized by at least two of the following: radiologic progression of fibrosis on high-resolution computed tomography (HRCT), progressive deterioration of respiratory complaints, and a decline in pulmonary function despite ongoing treatment efforts [3]. This phenotype is associated with a more severe clinical course, including reduced survival and higher morbidity compared to patients with non-progressive ILD [4]. As such, early recognition and intervention are essential to prevent irreversible pulmonary damage. Recent therapeutic interest has increased following the approval of nintedanib for idiopathic pulmonary fibrosis (IPF), prompting exploration of its efficacy in patients with CTD-ILD exhibiting a PF-ILD phenotype. Irrespective of the specific connective tissue disease, individuals with CTD-ILD are susceptible to progressive fibrosis through overlapping fibrotic mechanisms [5,6]. A systematic review indicates that approximately 20% to 40% of CTD-ILD patients experience disease progression consistent with PF-ILD [7,8,9]. Among CTD subtypes, systemic sclerosis-associated ILD (SSc-ILD) and rheumatoid arthritis-associated ILD (RA-ILD) most frequently present with this fibrosing phenotype [3]. In one cohort study, around 25% of patients with CTD developed progressive ILD within two years [10]. Another study reported PF-ILD in about one-third of their SSc-ILD cohort [11]. For RA-ILD, a retrospective analysis revealed that nearly half of the patients exhibited features consistent with PF-ILD [12], while the European PERSEID study identified progression in 38% of such patients [13]. Moreover, in idiopathic inflammatory myopathy-associated ILD (IIM-ILD), about 20% were classified as having a progressive phenotype in a cohort of 78 individuals [14]. Despite these findings, current data on the incidence and predictive factors of PF-ILD across CTD subtypes remain limited, underscoring the need for further longitudinal research.

### 3.1. Genetic and Epigenetic Contributions to PF-ILD

Recent studies have revealed the associated genetic mechanisms of PF-ILD. Alterations in telomere-related genes—TERT, TERC, RTEL1, PARN, TINF2, NAF1, and DKC1—have been linked to conditions such as IPF, iNSIP, RA-ILD, acute interstitial pneumonia, organizing pneumonia, and chronic hypersensitivity pneumonitis [15,16]. Telomeres, which safeguard chromosomal ends, are maintained by telomerase complexes. Dysfunction in this system impairs epithelial regeneration following injury and may drive fibrosis [17]. In animal models, defective telomere maintenance results in spontaneous or injury-accelerated pulmonary fibrosis, and targeted deletion of telomere-stabilizing proteins like TRF1 in alveolar type II cells induces fibrotic changes [18]. Conversely, telomerase reactivation has shown anti-fibrotic potential in preclinical settings [19].

Clinically, short telomere length is frequently observed in ILD patients, particularly in IPF, and correlates with impaired immune responses. In RA-ILD, mutations in RTEL1 and TERT have been associated with shortened telomeres and earlier disease onset [20].

Beyond telomere biology, a common promoter variant in the MUC5B gene (rs35705950) has been identified as a risk factor for IPF and has also been implicated in RA-ILD, hypersensitivity pneumonitis, and IPAF, but not in SSc-ILD, myositis-ILD, or sarcoidosis, illustrating genetic variability across ILD subtypes [21,22]. Although most genetic data come from IPF, variants in other genes have been described in RA-ILD and chronic hypersensitivity pneumonitis [23].

Additionally, epigenetic mechanisms such as DNA methylation, histone modification, and non-coding RNA activity play critical roles in regulating gene expression and may mediate environmental exposures like cigarette smoke or air pollutants or aging-related fibrotic responses in ILDs including IPF and RA-ILD [24,25].

### 3.2. Biological Mechanisms of PF-ILD in CTD-ILD

The mechanism of progressive fibrosis in CTD-ILD involves a complex interplay of inflammation, damage, and repair processes. These processes lead to aberrant lung remodeling and scarring, ultimately resulting in reduced lung function and capacity. Fibrosis is generally characterized by tissue overgrowth, stiffness, and/or scarring due to excessive accumulation of extracellular matrix components (ECMs), particularly collagen [26]. Loss of alveolar function due to persistent fibrosis leads to respiratory failure and premature death. In addition to epithelial lung damage, cellular or humoral autoimmunities, endothelial dysfunction, granuloma formation, or alveolar macrophage activation may occur. After an injury, a wound-healing response is induced. And the primary cellular mediator of fibrosis is the collagen-secreting myofibroblast.

The development of fibrotic ILDs is closely linked to the coordinated involvement of both innate and adaptive immune responses, encompassing a wide spectrum of cell-mediated and humoral mechanisms. Studies using animal models have clarified the functional heterogeneity among T-cell subtypes: Th2 and Th17 cells have emerged as potent inducers of fibrosis, while Th1, Th22, and γδ T cells exhibit antifibrotic activity. Particularly, PD-1+ CD4+ T cells, which are accessible via existing immunotherapies, have shown profibrotic effects in preclinical models of pulmonary fibrosis, including those relevant to IPF [27].

B cells have also been implicated in fibrogenesis, within the innate immune compartment, macrophages and neutrophils contribute to fibrosis through the production of cytokines such as TGF-β, PDGF, and IL-6, promoting fibroblast activation and ECM accumulation [28]. The profibrotic Macrophage (M2a) phenotype, which secretes CCL22, PDGF-BB, and IL-6. Neutrophils contribute to fibrotic progression through enzymes such as elastase and matrix metalloproteinases (MMPs), which remodel the ECM and activate latent TGF-β [29].

Multiple soluble immune mediators also drive fibrosis. IL-13 facilitates the transition of fibroblasts into myofibroblasts via JNK signaling, while IL-17 synergizes with TGF-β to promote fibrotic pathways. TGF-β, a master regulator of fibrosis, promotes epithelial-to-mesenchymal transition (EMT) and drives fibroblast activation through both canonical and non-canonical pathways, including MAPK, ERK, and PI3K/Akt signaling. Additionally, PDGF enhances ECM gene expression in fibroblasts, while CCL2 facilitates fibrocyte recruitment and monocyte chemotaxis.

Despite these associations, the mechanistic basis distinguishing progressive from non-progressive fibrosis across ILD subtypes remains poorly understood. Among ILDs, IPF and SSc-ILD have been most extensively studied mechanistically. Both display macrophage activation with M2 polarization and similar T-cell profiles, including increased Th2, Th22, and Th17 cells, and elevated CD4/CD8 ratios. However, B-cell phenotypes and chemokine expression patterns diverge between the diseases. In IPF, T-cell-derived cytokines such as IL-4, IL-5, IL-10, and IL-17 predominate, while in SSc-ILD, additional mediators like IL-6, IL-13, and IL-22 are elevated [30]. Notably, IL-6 is a critical driver in SSc pathogenesis, enhancing collagen synthesis, promoting myofibroblast formation, and suppressing matrix metalloproteinases [31]. Elevated serum IL-6 levels have been proposed as a biomarker of early progression in SSc-ILD patients with preserved lung function (forced vital capacity (FVC) > 70%) [32]. CXCL4 has also emerged as a potential marker for SSc-associated pulmonary fibrosis progression [33].

Moreover, lung explant studies from transplant recipients with progressive ILDs—including IPF, SSc-ILD, and others—have revealed elevated levels of PDGF, FGF-2, VEGF, and CSF-1, reinforcing the role of growth factors in the fibrotic cascade [34].

## 4. Serologic Biomarkers Associated with PF-ILD in CTD

Biomarkers offer valuable tools for understanding the mechanisms underlying CTD-ILD, aiding in both diagnosis and prognosis. Because disease trajectories in CTD-ILD are often unpredictable, identifying reliable serologic or molecular indicators of progression is a research priority. Because in many cases, serologic biomarkers reflect the molecular pathways in fibrosis and indicate early disease before overt fibrosis. Serological biomarkers are less invasive than bronchoscopy or lung biopsy, this section will only discuss various serologic biomarkers, including autoantibodies, cytokines, and protein markers that may aid in detecting progressive phenotypes in CTD-ILD. Serological predictive biomarkers for each CTD-ILD are summarized in Table 1.

### 4.1. SSc-ILD

The presence of anti-topoisomerase I (anti-Scl-70) antibodies is consistently correlated with a higher likelihood of developing interstitial lung disease (ILD) and a more aggressive clinical course in systemic sclerosis (SSc) patients, including faster pulmonary function decline [18,19]. A recent 2024 study conducted in China incorporated anti-Scl-70 seropositivity and reduced serum IgA levels into a prognostic model, identifying anti-Scl-70 as an independent marker for fibrosing ILD progression [20]. Comparable findings have been reported in independent cohorts from Spain and Australia, where both serologic and T-cell phenotype data reinforced its predictive value [21,22,23]. In a study by In one study, patients harboring anti-Scl-70 antibodies were recognized as a distinct SSc-ILD subgroup at elevated risk of progressive fibrosis, exhibiting significantly worse survival outcomes [11].

Additionally, a recent Australian cohort study identified C-X-C motif chemokine ligand 4 (CXCL4) as a potential biomarker of ILD progression in SSc. In an analysis involving 252 individuals with SSc-ILD, elevated CXCL4 levels were significantly associated with disease progression in univariable analysis (hazard ratio [HR]: 1.38, 95% confidence interval [CI]: 1.2–1.88; *p* = 0.039) [53]. Another chemokine, C-C motif ligand 18 (CCL18), was found to be associated with an increased risk of ILD progression or mortality in this patient population [35].

Krebs von den Lungen-6 (KL-6), a mucin-like glycoprotein secreted by alveolar type II epithelial cells, has been extensively studied in the context of CTD-ILDs and is known to reflect ongoing epithelial injury and fibrotic activity. Elevated KL-6 levels have been detected in SSc-ILD and are strongly associated with worsening pulmonary function and radiographic abnormalities [36,37]. Data from the GENISOS cohort revealed that patients with higher baseline KL-6 levels experienced a significantly faster annual decline in FVC, averaging an additional 7% loss per year [38].

Additional research has highlighted a panel of serum biomarkers linked to more severe fibrotic trajectories in SSc-ILD, including lower levels of fractional exhaled nitric oxide and interleukin (IL)-10, along with elevated concentrations of carbohydrate antigen 15.3 (CA15-3), C-reactive protein (CRP), and monocyte chemoattractant protein-1 (MCP-1) [54]. A broader summary also emphasized the prognostic utility of KL-6 and CRP, while CCL18 was reaffirmed as a particularly robust predictor of disease course. Other candidate biomarkers under investigation include matrix metalloproteinases (MMPs)-7 and -12, chemokines such as CCL2 and CXCL4, IL-6, chitinase-3-like protein 1 (YKL-40), and chitinase 1 [39]. Histological and immunological studies have additionally revealed extensive B-cell accumulation and M2 polarization of alveolar macrophages in SSc-ILD lungs. These alternatively activated macrophages, induced by cytokines like IL-4 and IL-10, secrete profibrotic mediators—such as CCL22, platelet-derived growth factor-BB (PDGF-BB), and IL-6—that drive fibrogenesis and interstitial remodeling [55]. Although IL-6 has been evaluated as a potential biomarker for disease progression, findings across studies remain inconclusive [31].

### 4.2. IIM-ILD

In a study, anti-melanoma differentiation-associated gene 5 (anti-MDA5) antibodies were significantly more frequent among patients with progressive fibrosing ILD (PF-ILD) compared to those without disease progression (29% vs. 6%, *p* = 0.03). Further research has confirmed a strong association between anti-MDA5 positivity and rapidly progressive ILD (RP-ILD), particularly in the context of dermatomyositis, with affected individuals demonstrating markedly poor survival outcomes [40]. A Japanese cohort study also found that anti-MDA5-positive idiopathic inflammatory myopathy (IIM) cases often progress to fulminant and fatal ILD, with an estimated 30–60% incidence and a six-fold increase in mortality risk compared to anti-MDA5-negative counterparts [41].

Anti-Ro52, an autoantibody targeting an intracellular ubiquitin ligase involved in antiviral defense, has also been investigated for its association with ILD in IIM. Patients who are anti-Ro52 positive exhibit a higher frequency of ILD manifestations [42,43]. While some systematic reviews have reported a potential link between anti-Ro52 and RP-ILD in myositis patients, the evidence remains inconclusive [44]. Nonetheless, more recent findings suggest that the presence of anti-Ro52 antibodies may be indicative of RP-ILD in IIM, highlighting its potential as a prognostic biomarker [45].

Analogous to systemic sclerosis-associated ILD (SSc-ILD), chemokine CCL18 has emerged as a candidate biomarker for fibrosing disease in IIM-ILD. In an investigation, elevated serum CCL18 concentrations were independently associated with both ILD diagnosis and progression in patients with IIM. Multivariate logistic regression analysis confirmed CCL18 as a significant predictor of PF-ILD, with an odds ratio of 1.006 (95% CI: 1.002–1.011, *p* = 0.005) [46]. Additionally, a recent review highlighted the prognostic value of various serological markers in IIM-ILD, including chitinase-3-like protein 1 (YKL-40) and a range of myositis-specific autoantibodies such as anti-aminoacyl-tRNA synthetase and anti-MDA5 [56]. Elevated interleukin-6 (IL-6) levels have also been associated with worse clinical outcomes in patients with anti-MDA5-positive dermatomyositis and RP-ILD, further reinforcing IL-6 as a potential prognostic indicator in this subgroup [47].

### 4.3. RA-ILD

A recent prospective cohort study monitored 136 individuals diagnosed with RA-ILD over a median follow-up of three years. Their analysis demonstrated that elevated baseline levels of serum biomarkers such as Krebs von den Lungen-6 (KL-6) and surfactant protein D (SP-D) were predictive of subsequent ILD progression. Notably, a one-year increase in KL-6 levels was most strongly associated with worsening ILD, with a hazard ratio (HR) of 2.00 (95% confidence interval [CI]: 1.29–3.11), even after adjusting for initial biomarker concentrations [50]. Supporting this, a systematic review identified multiple serum-based indicators—including anti-citrullinated protein antibodies (ACPAs) [48], KL-6 [51,52], matrix metalloproteinase-13 (MMP-13), and C-X-C motif chemokine ligand 11/interferon-inducible T cell alpha chemoattractant (CXCL11/I-TAC)—as being associated with progressive disease in RA-ILD [49].

Histological studies of RA-ILD lung tissue commonly reveal marked infiltration of CD4 + T cells and hyperplastic expansion of follicular B cells [57]. These immune cell populations may promote fibrogenic pathways through both cellular and humoral mechanisms. Among soluble mediators, interleukin (IL)-13 and IL-17 play key roles, with IL-13 enhancing the transformation of fibroblasts into myofibroblasts, and IL-17 facilitating fibroblast proliferation. In RA-ILD cases with a usual interstitial pneumonia (UIP) pattern, the Janus kinase 2 (JAK2)/signal transducer and activator of transcription 3 (STAT3) pathway has been implicated in mediating transforming growth factor-beta (TGF-β)-driven myofibroblast activation, thus playing a central role in the fibrotic remodeling of the lung parenchyma [58].

## 5. Treatment Approaches of PF-ILD in CTD

Therapeutic strategies for CTD-ILD are increasingly incorporating antifibrotic agents, guided in part by biomarker data. Compounds like nintedanib and pirfenidone, initially developed for IPF, are now being evaluated for their role in fibrosing CTD-ILD. Ongoing studies aim to identify which patient subsets benefit most from these therapies, potentially leading to biomarker-driven treatment customization. Current treatment recommendations are generally based on baseline CTD, but new treatment guidelines based on the progressive phenotype appear warranted. In this section, we will focus on the newly emerging anti-fibrosing treatments in addition to the conventional treatments such as existing immunosuppressants such as cyclophosphamide (CYC) and mycophenolate mofetil (MMF). A summary of emerging treatments of CTD-ILD is presented in Table 2.

### 5.1. SSc-ILD

#### 5.1.1. Immunosuppressants

Evidence supporting CYC in SSc-ILD originates from the Scleroderma Lung Study I (SLS I), a landmark randomized, double-blind, multicenter trial. Participants received oral CYC at 2 mg/kg/day for one year followed by 12 months of observation. The study found a modest but statistically significant benefit in FVC (mean difference of 2.53%) favoring CYC at the 12-month. However, by 24 months, this difference in FVC% was no longer observed between the treatment and placebo groups. Additionally, the transitional dyspnea index improved in patients receiving CYC, contrasting with a decline in those on placebo (*p* < 0.001) [59]. Despite its demonstrated efficacy, the clinical utility of CYC is constrained by its toxicity profile, which limits its widespread application in routine practice.

Scleroderma Lung Study II (SLS II) compared MMF (1500 mg twice daily for 24 months) with a 12-month course of oral CYC followed by 12 months of placebo. Both agents led to significant gains in lung function, However, there was no statistically significant difference in the primary outcome measure, FVC at 24 months [60].

The focuSSced trial—a phase 3 multicenter, double-blind, placebo-controlled study—randomized 210 participants with early diffuse cutaneous systemic sclerosis (dcSSc) to receive subcutaneous tocilizumab or placebo. While the primary endpoint (skin involvement) was not met (*p* = 0.10), patients receiving tocilizumab experienced less FVC decline (−0.1% vs. −6.3%, *p* < 0.0001) [61]. HRCT imaging revealed the progression of fibrotic changes in the placebo group (*p* < 0.001), whereas such progression was not observed in the tocilizumab group (*p* = 0.12) [62]. Long-term follow-up at week 96 confirmed FVC stabilization in tocilizumab-treated patients and improvement in those switched from placebo [63].

The DESIRES trial in Japan randomized patients to rituximab (375 mg/m² weekly × 4) or placebo. Among 48 participants with ILD, rituximab recipients showed a 2.96% higher predicted FVC at 24 weeks (*p* = 0.044) [64]. Longitudinal data from 29 patients followed up to 96 weeks indicated cumulative improvement after repeated RTX courses, with significant FVC gains after the third cycle (median change 1.85%, *p* < 0.01) [65].

#### 5.1.2. Antifibrotic Agents

Nintedanib is a broad-spectrum tyrosine kinase inhibitor that exerts antifibrotic effects by targeting several profibrotic pathways, particularly those involving platelet-derived growth factor (PDGF), vascular endothelial growth factor (VEGF), and fibroblast growth factor (FGF) receptors [43]. Initially approved in 2014 for IPF [44], the therapeutic profile of nintedanib has since been expanded based on preclinical studies, which demonstrated its anti-inflammatory, antifibrotic, and antiangiogenic effects in systemic sclerosis (SSc) models and other forms of fibrosing ILD [45,46]. The pivotal SENSCIS trial, a randomized, double-blind, placebo-controlled phase III study, was instrumental in demonstrating that nintedanib significantly attenuated the rate of decline in FVC in patients with SSc-ILD. This led to its regulatory approval for SSc-ILD in 2019 [50,51,52].

Subsequent evidence from the INBUILD trial, another phase III, multicenter, randomized, double-blind, placebo-controlled study, evaluated nintedanib in a cohort of patients with PF-ILDs unrelated to IPF [66]. Over a 52-week treatment period, individuals receiving nintedanib experienced a significantly reduced rate of FVC decline compared to those in the placebo arm [67]. A post hoc subgroup analysis revealed consistent efficacy across a range of ILD subtypes, including CTD-ILD, reinforcing its therapeutic utility beyond IPF [68]. As a result, in 2020, nintedanib was granted extended approval for the treatment of PF-ILDs regardless of etiology. Although the INBUILD trial included a substantial number of participants with SSc-ILD and RA-ILD, representation of other CTD-related ILDs was limited, highlighting the need for more comprehensive studies across the full spectrum of CTD-ILDs. Furthermore, a subgroup analysis from the SENSCIS trial explored the safety of combining nintedanib with MMF, finding that the combination was generally well tolerated and did not result in a higher incidence of adverse events compared to MMF alone [69].

Pirfenidone is an orally administered antifibrotic medication that functions by suppressing fibroblast proliferation and collagen synthesis, thereby mitigating the development of fibrotic tissue [70]. Clinical trials have demonstrated that pirfenidone effectively slows FVC decline in patients with IPF as well as those with unclassifiable progressive fibrosing ILD [71]. A small, double-blind, randomized trial evaluated pirfenidone (2400 mg/day) in patients with progressive SSc-ILD who had not previously received biologics, assigning 17 patients each to pirfenidone or placebo. The study found no significant difference in the primary outcome of FVC change between groups (*p* = 0.33) [72]. Due to its limited sample size, the trial lacked the power to draw definitive conclusions.

### 5.2. IIM-ILD

#### 5.2.1. Immunosuppressants

In one large single-center retrospective analysis involving 110 patients, 66 received azathioprine (AZA) and 44 received MMF. Baseline FVC and DLCO were lower in the AZA group, but both groups showed improvement in FVC and tapering of prednisone. After adjusting for variables such as sex, age, antisynthetase antibody status, and smoking, no significant difference in treatment effect was found. However, adverse events were more frequent with AZA than with MMF [73].

Although randomized controlled trials are lacking, RTX is often used in IIM-ILD, particularly in treatment-refractory cases. A phase 2 open-label multicenter trial evaluated rituximab (1000 mg on day 0, day 15, and month 6) in 10 patients with antisynthetase-positive refractory IIM. All had ILD and were permitted concurrent therapies including IV steroids, immunosuppressants, IVIG, or plasma exchange. Dyspnea improved in some cases; FVC increased in four patients, remained stable in five, and declined in one [74]. A retrospective case-control study compared RTX (n = 28) and CYC (n = 34) in patients with antisynthetase syndrome-ILD. RTX was given as two 1000 mg infusions 15 days apart, with redosing at 6 months. While 6-month progression-free survival was similar (92% RTX vs. 85% CYC), RTX showed superior progression-free survival at 2 years (HR 0.263, CI: 0.094–0.732, *p* = 0.011). Median FVC in the RTX group rose from 64% at baseline to 92% at 12 months and 90% at 2 years (*p* = 0.0005). A systematic review of 17 studies including 35 anti-MDA5-positive ILD patients reported a 71.4% overall response rate to RTX based on imaging or pulmonary function tests, with comparable efficacy in both rapidly progressive and chronic ILD subtypes [75].

Calcineurin inhibitors, including tacrolimus and cyclosporine, are frequently used in cases of refractory IIM-ILD or as part of combination regimens for early, severe disease [76]. A prospective, multicenter, open-label randomized trial compared prednisolone with tacrolimus (n = 30) versus prednisolone with cyclosporine (n = 28) in patients with IIM-ILD. At 52 weeks, progression-free survival was higher in the tacrolimus group (87%) compared to the cyclosporine group (71%), and both groups experienced an increase in FVC [77].

Combination immunosuppressive therapy has been suggested as a first-line approach for patients with anti-MDA5-positive dermatomyositis [78]. A prospective, multicenter, open-label study in Japan evaluated 29 patients with ILD who received initial triple therapy consisting of prednisolone (1 mg/kg/day), intravenous CYC, and tacrolimus. Plasmapheresis was permitted in cases of disease progression despite treatment. Outcomes were compared to a historical “step-up” treatment cohort. At six months, the combination group had significantly higher survival (89% vs. 33%, *p* < 0.0001). Improvements in FVC (*p* < 0.0125) and HRCT scores (*p* < 0.0167) were also noted in the combination group [79].

Initial evidence supporting the use of tofacitinib in anti-MDA5-positive clinically amyopathic dermatomyositis (CADM) came from case reports and small series involving patients unresponsive to standard combination therapy. In the largest series, five high-risk patients (e.g., ferritin >1000 ng/mL, diffuse ground-glass opacities, worsening infiltrates) were treated with tofacitinib 10 mg daily. Three survived, suggesting improved outcomes compared to historical controls [80]. Subsequent prospective studies were conducted. At a single center in China, 18 patients received tofacitinib 5 mg twice daily and were compared to 32 historical controls; 6-month survival was significantly better in the tofacitinib group (100% vs. 78%, *p* = 0.04) [81]. Another study of 15 newly diagnosed anti-MDA5-positive CADM patients reported a 67% response rate after 6 months of tofacitinib, with improvement in DLCO but not FVC (*p* = 0.009) [82]. A separate case series of six patients with RP-ILD who failed triple therapy received tofacitinib 10–20 mg/day. Four improved, but two died, and all experienced infectious complications, including CMV, pulmonary aspergillosis, and herpesvirus infections [83].

Evidence supporting intravenous immunoglobulin (IVIG) use in IIM-ILD is largely limited to case reports and series, and it is not routinely recommended. However, some reports suggest the potential benefit of IVIG in both antisynthetase syndrome and anti-MDA5-positive ILD. In a single-center retrospective study of 11 patients with refractory ASS-ILD, those recently treated with rituximab or receiving IVIG for nonpulmonary reasons were excluded. Over a 25-month period, mean FVC and DLCO improved (*p* = 0.048 and 0.022), with seven patients showing a >10% FVC increase. Steroid doses also declined significantly (from 20 mg to <10 mg, *p* < 0.001) [84]. Another retrospective study in China evaluated patients with new-onset anti-MDA5-positive rapidly progressive ILD. Compared to those who did not receive IVIG (n = 17), the IVIG group (n = 31) had lower 6-month mortality (22.6% vs. 52.9%, *p* = 0.033) and higher remission at 3 months (71% vs. 41.2%, *p* = 0.044) [85].

#### 5.2.2. Antifibrotic Agents

In the context of IIM-ILD, which is often characterized by a more inflammatory than fibrotic phenotype, a subset of patients may still progress to early fibrosis. Retrospective analyses involving small case series have reported favorable responses to antifibrotic therapies, including nintedanib and pirfenidone, in patients with fibrotic IIM-ILD, offering preliminary support for their potential role in this population and aligning with outcomes seen in the INBUILD study [86,87]. A prospective study involving 27 patients with IIM-ILD and disease onset within six months, particularly those diagnosed with amyopathic dermatomyositis, assessed the addition of pirfenidone to standard immunosuppressive therapy. When compared with a historical control group not treated with pirfenidone, patients with subacute ILD (3–6 months duration) demonstrated a significant reduction in mortality. However, among those with acute ILD (disease duration less than 3 months), no statistically significant survival benefit was observed. Radiographic findings were comparable between the groups, and FVC data were not assessable due to incomplete documentation. Among the surviving patients, three discontinued pirfenidone due to drug-related adverse events [86].

### 5.3. RA-ILD

#### 5.3.1. Immunosuppressants

Immunosuppressive agents such as mycophenolate mofetil (MMF), azathioprine, and rituximab are widely used as first-line therapies for RA-associated interstitial lung disease (RA-ILD). Evidence from a large multicenter retrospective cohort study involving 212 patients showed that immunosuppressive therapy resulted in a significant improvement in FVC (+3.90%, *p* ≤ 0.001) and DLCO (+4.53%, *p* ≤ 0.001) compared to the pretreatment trajectory [88]. Additional retrospective studies have supported RTX’s role in RA-ILD, showing its potential to stabilize or improve lung function and possibly reduce overall mortality [89]. Recent data suggest abatacept may be effective in RA-ILD. In a Spanish multicenter observational study involving 263 patients, outcomes after a median of 12 months showed that 92% had stable or improved dyspnea, 88% had stable or improved FVC, 91% had stable or improved DLCO, and 77% showed no worsening on chest HRCT. Additionally, patients experienced a significant reduction in disease activity as measured by the DAS28 score [90].

#### 5.3.2. Antifibrotic Agents

The TRAIL1 trial was a phase II, multicenter, randomized, double-blind, placebo-controlled study that evaluated pirfenidone in RA-ILD. Results indicated a slower annual decline in FVC among patients receiving pirfenidone (66 mL/year) compared to those receiving placebo (146 mL/year). Notably, in patients with a usual interstitial pneumonia (UIP) pattern, the difference was even more marked—43 mL decline in the pirfenidone group versus 169 mL in the placebo group [91]. Additionally, pirfenidone lowered the concentrations of inflammatory cytokines, including interleukin-6 and tumor necrosis factor-alpha, both of which are implicated in RA pathogenesis [92]. In vitro studies, further confirmed that pirfenidone inhibits the fibroblast-to-myofibroblast differentiation in lung fibroblasts derived from RA-ILD patients [93].

## 6. Challenges and Future Directions

Despite advances in the identification of potential biomarkers for connective tissue disease-associated interstitial lung disease (CTD-ILD), several important obstacles remain. One of the key limitations is the biological and clinical heterogeneity of CTD-ILDs, which makes it difficult to establish biomarkers that are broadly applicable across all subtypes. Additionally, while numerous candidate biomarkers have shown promise in preliminary studies, their incorporation into routine clinical practice demands rigorous validation through large-scale, longitudinal, and multicenter trials.

To address these limitations, future research should focus on the application of integrative multi-omics strategies—including genomics, proteomics, and transcriptomics—to uncover novel molecular signatures that can more precisely define disease endotypes and guide personalized treatment approaches. Such methodologies may enable a more accurate stratification of patients and improve prognostication and therapeutic decision-making.

Several investigational therapies are currently under evaluation for SSc-ILD, reflecting the growing interest in targeted treatment strategies. These include vixarelimab (NCT05785624) [94], MK 7240 007/PRA023 (NCT05270668) [95], belimumab (NCT05878717) [96], and anifrolumab (NCT05925803) [97], all of which are being studied in clinical trials aiming to assess their efficacy and safety in SSc-ILD. In contrast, there remains a relative paucity of well-powered, controlled studies assessing immunosuppressive and antifibrotic therapies specifically in IIM-ILD.

One notable trial in this area is the Myositis Interstitial Lung Disease Nintedanib Trial (MINT), which aims to evaluate the effectiveness of nintedanib in patients with progressive fibrosing ILD associated with IIM. Although patient enrollment for MINT (NCT05799755) has concluded, trial results have not yet been published [98]. The outcomes of such studies will be critical for establishing evidence-based treatment paradigms and identifying which subsets of CTD-ILD patients are most likely to benefit from antifibrotic therapies.

## 7. Conclusions

Connective tissue disease-associated ILD is a heterogeneous condition in which a subset of patients develops progressive fibrosing lung disease. Biomarkers hold the potential to transform the diagnosis, prognosis, and treatment of CTD-ILD by enhancing our understanding of the pathogenesis. Although several candidate biomarkers—such as KL-6, SP-D, CXCL4, and anti-MDA5—show potential to predict progression or treatment response, most are limited by disease specificity and lack of prospective validation. With ongoing research into the molecular mechanisms underlying CTD-ILD, more precise and personalized strategies for managing this complex and diverse disease spectrum are likely in the future. Antifibrotic agents such as nintedanib and pirfenidone, and immunomodulators including tocilizumab and rituximab, offer promising therapeutic options for selected patients. However, current evidence is fragmented, and biomarker-guided treatment strategies remain undeveloped. Future research should focus on validating integrated biomarker panels and tailoring therapy based on molecular endotypes to improve patient outcomes.

## Figures and Tables

**Table 1:** Predictive biomarkers associated with progressive fibrosing CTD-ILD.

CTD Type	Biomarker	Association with PF-ILD	Reference(s)
**SSc**	Anti-Scl-70 (anti-topoisomerase I)	Associated with ILD risk and faster FVC decline	[20,21,22,23]
	IL-6	Linked to fibrosis progression; results vary across studies	[22,30,31,32]
	CCL18	Correlates with ILD progression and mortality	[35]
	KL-6	Associated with ILD severity and progression	[36,37,38]
	MMP-7, MMP-12	Associated with lung remodeling and fibrosis	[39]
**IIM**	Anti-MDA5	Predicts RP-ILD in dermatomyositis and poor prognosis	[40,41]
	Anti-Ro52	Associated with ILD, possibly RP-ILD	[42,43,44,45]
	CCL18	Independent predictor of PF-ILD in IIM	[46]
	IL-6	Associated with poor outcomes in anti-MDA5+ cases	[47]
**RA**	ACPA	Associated with RA-ILD progression	[48]
	CXCL11 (I-TAC)	Elevated in progressive RA-ILD	[49]
	KL-6	Associated with ILD severity and progression	[50,51,52]
	MMP-13	Associated with lung remodeling and fibrosis	[49]

CTD, connective tissue disease; PF, progressive fibrosing; ILD, interstitial lung disease; SSc, systemic sclerosis; FVC, Forced Vital Capacity; IL-6, interleukin-6; CCL18, C-C motif chemokine ligand 18; KL-6, Krebs von den Lungen-6;MMP, matrix metallopeptidase; IIM, idiopathic inflammatory myopathy; RP-ILD, Rapidly progressive interstitial lung disease; anti-MDA5, anti-melanoma differentiation-associated gene 5; ACPA, anti-citrullinated protein antibody; RA, rheumatoid arthritis; CXCL11, C-X-C motif chemokine ligand 11.

**Table 2:** Existing and emerging therapies for PF-ILD in CTD.

Drug Name	Mechanism of Action	Clinical Stage
Nintedanib	Tyrosine kinase inhibitor targeting PDGF, FGF, and VEGF receptors	Approved (IPF, SSc-ILD, PF-ILD)
Pirfenidone	Antifibrotic and anti-inflammatory effects via TGF-β inhibition	Approved (IPF; off-label for other ILDs)
Vixarelimab	Anti-oncostatin M receptor β antibody	Phase II/III (SSc-ILD)
Abatacept	T-cell co-stimulation modulator (CTLA-4 agonist)	Phase III (RA-ILD, CTD-ILD)
Rituximab	CD20+ B-cell depletion	Used off-label; Phase III in CTD-ILD
Anifrolumab	Type I IFN receptor antagonist	Phase III (SLE, SSc-ILD)
Belimumab	Anti-BAFF monoclonal antibody	Phase III (SSc-ILD)
Tocilizumab	IL-6 receptor antagonist	Approved (RA); off-label use in ILD
Lenabasum	Cannabinoid receptor *2* agonist with anti-inflammatory effects	Phase II (SSc-ILD)
Iberdomide	Cereblon E3 ligase modulator affecting immune signaling	Phase I/II (SSc-ILD)

IPF, Idiopathic Pulmonary Fibrosis; SSc, Systemic Sclerosis; ILD, Interstitial Lung Disease; PF, Progressive Fibrosing; PDGF, Platelet-Derived Growth Factor; FGF, Fibroblast Growth Factor; VEGF, Vascular Endothelial Growth Factor; TGF, Transforming Growth Factor; CTLA-4, Cytotoxic T-Lymphocyte-Associated Protein 4; RA, Rheumatoid Arthritis; CTD, Connective Tissue Disease; SLE, Systemic Lupus Erythematosus; IFN, Interferon; BAFF, B Cell Activating Factor; IL, Interleukin.

## Data Availability

No new data were created or analyzed in this study. Data sharing is not applicable to this article.

## Abbreviations

PDGF, platelet-derived growth factor; MMF, mycophenolate mofetil; FVC, forced vital capacity; SSc, systemic sclerosis; ILD, interstitial lung disease; PF-ILD, progressive fibrosing ILD; CTD, connective tissue disease; RA, rheumatoid arthritis.

## References

[1] Cottin V. (2016). Idiopathic interstitial pneumonias with connective tissue diseases features: A review. Respirology.

[2] Copeland C.R., Lancaster L.H. (2021). Management of progressive fibrosing interstitial lung diseases (PF-ILD). Front. Med..

[3] Raghu G., Remy-Jardin M., Richeldi L., Thomson C.C., Inoue Y., Johkoh T., Kreuter M., Lynch D.A., Maher T.M., Martinez F.J. (2022). Idiopathic pulmonary fibrosis (an update) and progressive pulmonary fibrosis in adults: An official ATS/ERS/JRS/ALAT clinical practice guideline. Am. J. Respir. Crit. Care Med..

[4] Simpson T., Barratt S.L., Beirne P., Chaudhuri N., Crawshaw A., Crowley L.E., Fletcher S., Gibbons M.A., Hallchurch P., Horgan L. (2021). The burden of progressive fibrotic interstitial lung disease across the UK. Eur. Respir. J..

[5] Zanatta E., Huscher D., Ortolan A., Avouac J., Airò P., Balbir-Gurman A., Siegert E., Matucci Cerinic M., Cozzi F., Riemekasten G. (2022). Phenotype of limited cutaneous systemic sclerosis patients with positive anti-topoisomerase I antibodies: Data from the EUSTAR cohort. Rheumatology.

[6] Cottin V. (2019). Treatment of progressive fibrosing interstitial lung diseases: A milestone in the management of interstitial lung diseases. Eur. Respir. Rev..

[7] Zamora-Legoff J.A., Krause M.L., Crowson C.S., Ryu J.H., Matteson E.L. (2017). Progressive Decline of Lung Function in Rheumatoid Arthritis-Associated Interstitial Lung Disease. Arthritis Rheumatol..

[8] Hoffmann-Vold A.M., Aalokken T.M., Lund M.B., Garen T., Midtvedt O., Brunborg C., Gran J.T., Molberg O. (2015). Predictive value of serial high-resolution computed tomography analyses and concurrent lung function tests in systemic sclerosis. Arthritis Rheumatol..

[9] Marie I., Hatron P.Y., Dominique S., Cherin P., Mouthon L., Menard J.F. (2011). Short-term and long-term outcomes of interstitial lung disease in polymyositis and dermatomyositis: A series of 107 patients. Arthritis Rheum..

[10] Takei R., Brown K.K., Yamano Y., Kataoka K., Yokoyama T., Matsuda T., Kimura T., Suzuki A., Furukawa T., Fukuoka J. (2022). Prevalence and prognosis of chronic fibrosing interstitial lung diseases with a progressive phenotype. Respirology.

[11] Morrisroe K., Hansen D., Stevens W., Ross L., Sahhar J., Ngian G.-S., Hill C.L., Host L., Walker J., Proudman S. (2024). Progressive pulmonary fibrosis and its impact on survival in systemic sclerosis-related interstitial lung disease. Rheumatology.

[12] Denis A., Henket M., Ernst M., Maes N., Thys M., Regnier C., Malaise O., Frix A.-N., Gester F., Desir C. (2022). Progressive fibrosing interstitial lung disease in rheumatoid arthritis: A retrospective study. Front. Med..

[13] Hilberg O., Hoffmann-Vold A.-M., Smith V., Bouros D., Kilpeläinen M., Guiot J., Morais A., Clemente S., Daniil Z., Papakosta D. (2022). Epidemiology of interstitial lung diseases and their progressive-fibrosing behaviour in six European countries. ERJ Open Res..

[14] Hambly N., Farooqi M.M., Dvorkin-Gheva A., Donohoe K., Garlick K., Scallan C., Chong S.G., MacIsaac S., Assayag D., Johannson K.A. (2022). Prevalence and characteristics of progressive fibrosing interstitial lung disease in a prospective registry. Eur. Respir. J..

[15] Arish N., Petukhov D., Wallach-Dayan S.B. (2019). The role of telomerase and telomeres in interstitial lung diseases: From molecules to clinical implications. Int. J. Mol. Sci..

[16] Bouros D., Tzouvelekis A. (2019). Telomeropathy Chronic Hypersensitivity Pneumonitis. Am. Thorac. Soc..

[17] Alder J.K., Barkauskas C.E., Limjunyawong N., Stanley S.E., Kembou F., Tuder R.M., Hogan B.L., Mitzner W., Armanios M. (2015). Telomere dysfunction causes alveolar stem cell failure. Proc. Natl. Acad. Sci. USA.

[18] Naikawadi R.P., Disayabutr S., Mallavia B., Donne M.L., Green G., La J.L., Rock J.R., Looney M.R., Wolters P.J. (2016). Telomere dysfunction in alveolar epithelial cells causes lung remodeling and fibrosis. JCI Insight.

[19] Povedano Selfa J.M., Martinez Rodriguez P., Serrano Ruiz R., Tejera A., Gomez Lopez G., Bobadilla M., Flores J.M., Bosch F., Blasco MA M.A. (2018). Therapeutic effects of telomerase in mice with pulmonary fibrosis induced by damage to the lungs and short telomeres. eLife.

[20] Juge P.-A., Borie R., Kannengiesser C., Gazal S., Revy P., Wemeau-Stervinou L., Debray M.-P., Ottaviani S., Marchand-Adam S., Nathan N. (2017). Shared genetic predisposition in rheumatoid arthritis-interstitial lung disease and familial pulmonary fibrosis. Eur. Respir. J..

[21] Jiang H., Hu Y., Shang L., Li Y., Yang L., Chen Y. (2015). Association between MUC5B polymorphism and susceptibility and severity of idiopathic pulmonary fibrosis. Int. J. Clin. Exp. Pathol..

[22] Johnson C., Rosen P., Lloyd T., Horton M., Christopher-Stine L., Oddis C.V., Mammen A.L., Danoff S.K. (2017). Exploration of the MUC5B promoter variant and ILD risk in patients with autoimmune myositis. Respir. Med..

[23] Adegunsoye A., Vij R., Noth I. (2019). Integrating genomics into management of fibrotic interstitial lung disease. Chest.

[24] Tzouvelekis A., Kaminski N. (2015). Epigenetics in idiopathic pulmonary fibrosis. Biochem. Cell Biol..

[25] Gulati S., Thannickal V.J. (2019). The aging lung and idiopathic pulmonary fibrosis. Am. J. Med. Sci..

[26] Ta W. (2008). Cellular and molecular mechanisms of fibrosis. J. Pathol..

[27] Celada L.J., Kropski J.A., Herazo-Maya J.D., Luo W., Creecy A., Abad A.T., Chioma O.S., Lee G., Hassell N.E., Shaginurova G.I. (2018). PD-1 up-regulation on CD4+ T cells promotes pulmonary fibrosis through STAT3-mediated IL-17A and TGF-β1 production. Sci. Transl. Med..

[28] Bellamri N., Morzadec C., Joannes A., Lecureur V., Wollin L., Jouneau S., Vernhet L. (2019). Alteration of human macrophage phenotypes by the anti-fibrotic drug nintedanib. Int. Immunopharmacol..

[29] Kolahian S., Fernandez I.E., Eickelberg O., Hartl D. (2016). Immune mechanisms in pulmonary fibrosis. Am. J. Respir. Cell Mol. Biol..

[30] Bagnato G., Harari S. (2015). Cellular interactions in the pathogenesis of interstitial lung diseases. Eur. Respir. Rev..

[31] Bonhomme O., André B., Gester F., de Seny D., Moermans C., Struman I., Louis R., Malaise M., Guiot J. (2019). Biomarkers in systemic sclerosis-associated interstitial lung disease: Review of the literature. Rheumatology.

[32] De Lauretis A., Sestini P., Pantelidis P., Hoyles R., Hansell D.M., Goh N.S., Zappala C.J., Visca D., Maher T.M., Denton C.P. (2013). Serum interleukin 6 is predictive of early functional decline and mortality in interstitial lung disease associated with systemic sclerosis. J. Rheumatol..

[33] van Bon L., Affandi A.J., Broen J., Christmann R.B., Marijnissen R.J., Stawski L., Farina G.A., Stifano G., Mathes A.L., Cossu M. (2014). Proteome-wide analysis and CXCL4 as a biomarker in systemic sclerosis. New Engl. J. Med..

[34] Hoffmann-Vold A.-M., Weigt S.S., Saggar R., Palchevskiy V., Volkmann E.R., Liang L.L., Ross D., Ardehali A., Lynch J.P., Belperio J.A. (2019). Endotype–phenotyping may predict a treatment response in progressive fibrosing interstitial lung disease. EBioMedicine.

[35] Distler O., Assassi S., Cottin V., Cutolo M., Danoff S.K., Denton C.P., Distler J.H., Hoffmann-Vold A.-M., Johnson S.R., Ladner U.M. (2020). Predictors of progression in systemic sclerosis patients with interstitial lung disease. Eur. Respir. J..

[36] Lee J.S., Lee E.Y., Ha Y.-J., Kang E.H., Lee Y.J., Song Y.W. (2019). Serum KL-6 levels reflect the severity of interstitial lung disease associated with connective tissue disease. Arthritis Res. Ther..

[37] Hant F.N., Ludwicka-Bradley A., Wang H.-J., Li N., Elashoff R., Tashkin D.P., Silver R.M. (2009). Surfactant protein D and KL-6 as serum biomarkers of interstitial lung disease in patients with scleroderma. J. Rheumatol..

[38] Salazar G.A., Kuwana M., Wu M., Estrada-Y-Martin R.M., Ying J., Charles J., Mayes M.D., Assassi S. (2018). KL-6 but not CCL-18 is a predictor of early progression in systemic sclerosis-related interstitial lung disease. J. Rheumatol..

[39] Khanna D., Tashkin D.P., Denton C.P., Renzoni E.A., Desai S.R., Varga J. (2020). Etiology, risk factors, and biomarkers in systemic sclerosis with interstitial lung disease. Am. J. Respir. Crit. Care Med..

[40] Moghadam-Kia S., Oddis C.V., Sato S., Kuwana M., Aggarwal R. (2016). Anti–melanoma differentiation–associated gene 5 is associated with rapidly progressive lung disease and poor survival in US patients with amyopathic and myopathic dermatomyositis. Arthritis Care Res..

[41] Sato S., Hirakata M., Kuwana M., Suwa A., Inada S., Mimori T., Nishikawa T., Oddis C.V., Ikeda Y. (2005). Autoantibodies to a 140-kd polypeptide, CADM-140, in Japanese patients with clinically amyopathic dermatomyositis. Arthritis Rheum..

[42] Nayebirad S., Mohamadi A., Yousefi-Koma H., Javadi M., Farahmand K., Atef-Yekta R., Tamartash Z., Jameie M., Mohammadzadegan A.M., Kavosi H. (2023). Association of anti-Ro52 autoantibody with interstitial lung disease in autoimmune diseases: A systematic review and meta-analysis. BMJ Open Respir. Res..

[43] Chiang H.-L., Tung C.-H., Huang K.-Y., Hsu B.-B., Wu C.-H., Hsu C.-W., Lu M.-C., Lai N.-S. (2021). Association between clinical phenotypes of dermatomyositis and polymyositis with myositis-specific antibodies and overlap systemic autoimmune diseases. Medicine.

[44] Ferreira J.P., Almeida I., Marinho A., Cerveira C., Vasconcelos C. (2012). Anti-ro52 antibodies and interstitial lung disease in connective tissue diseases excluding scleroderma. Int. Sch. Res. Not..

[45] Wang K., Tian Y., Liu S., Zhang Z., Shen L., Meng D., Li J. (2022). Risk factors and predictive model for dermatomyositis associated with rapidly progressive interstitial lung disease. Pharmacogenomics Pers. Med..

[46] Zanatta E., Martini A., Depascale R., Gamba A., Tonello M., Gatto M., Giraudo C., Balestro E., Doria A., Iaccarino L. (2023). CCL18 as a biomarker of interstitial lung disease (ILD) and progressive fibrosing ILD in patients with idiopathic inflammatory myopathies. Diagnostics.

[47] Niu Y., Liu S., Qiu Q., Fu D., Xiao Y., Liang L., Cui Y., Ye S., Xu H. (2024). Increased serum level of IL-6 predicts poor prognosis in anti-MDA5-positive dermatomyositis with rapidly progressive interstitial lung disease. Arthritis Res. Ther..

[48] Wang H.-F., Wang Y.-Y., Li Z.-Y., He P.-J., Liu S., Li Q.-S. (2024). The prevalence and risk factors of rheumatoid arthritis-associated interstitial lung disease: A systematic review and meta-analysis. Ann. Med..

[49] Chen J., Chen Y., Liu D., Lin Y., Zhu L., Song S., Hu Y., Liang T., Liu Y., Liu W. (2022). Predictors of long-term prognosis in rheumatoid arthritis-related interstitial lung disease. Sci. Rep..

[50] Chang S.H., Park Y.-B., McDermott G.C., Paudel M.L., Hayashi K., Ha Y.-J., Lee J.S., Kim M.U., Park C.H., Kim J.-W. (2025). Serum biomarkers of pulmonary damage and risk for progression of rheumatoid arthritis–associated interstitial lung disease. J. Rheumatol..

[51] Mochizuki T., Ikari K., Yano K., Sato M., Okazaki K. (2019). Long-term deterioration of interstitial lung disease in patients with rheumatoid arthritis treated with abatacept. Mod. Rheumatol..

[52] Fotoh D.S., Helal A., Rizk M.S., Esaily H.A. (2021). Serum Krebs von den Lungen-6 and lung ultrasound B lines as potential diagnostic and prognostic factors for rheumatoid arthritis–associated interstitial lung disease. Clin. Rheumatol..

[53] Parker M.J.S., Jee A.S., Hansen D., Proudman S., Youssef P., Kenna T.J., Stevens W., Nikpour M., Sahhar J., Corte T.J. (2024). Multiple serum biomarkers associate with mortality and interstitial lung disease progression in systemic sclerosis. Rheumatology.

[54] Perelas A., Silver R.M., Arrossi A.V., Highland K.B. (2020). Systemic sclerosis-associated interstitial lung disease. Lancet Respir. Med..

[55] Spagnolo P., Distler O., Ryerson C.J., Tzouvelekis A., Lee J.S., Bonella F., Bouros D., Hoffmann-Vold A.-M., Crestani B., Matteson E.L. (2021). Mechanisms of progressive fibrosis in connective tissue disease (CTD)-associated interstitial lung diseases (ILDs). Ann. Rheum. Dis..

[56] Cao H., Huang J., Chang J., Zhu Y., Liang J., Sun C., Lin J. (2023). Predictors of progression in idiopathic inflammatory myopathies with interstitial lung disease. J. Transl. Intern. Med..

[57] Gu B.-H., Madison M.C., Corry D., Kheradmand F. (2018). Matrix remodeling in chronic lung diseases. Matrix Biol..

[58] Wang S., Liu M., Li X., Zhang J., Wang F., Zhang C., Roden A., Ryu J.H., Warrington K.J., Sun J. (2022). Canonical and noncanonical regulatory roles for JAK2 in the pathogenesis of rheumatoid arthritis-associated interstitial lung disease and idiopathic pulmonary fibrosis. FASEB J..

[59] Tashkin D.P., Elashoff R., Clements P.J., Goldin J., Roth M.D., Furst D.E., Arriola E., Silver R., Strange C., Bolster M. (2006). Cyclophosphamide versus placebo in scleroderma lung disease. New Engl. J. Med..

[60] Jaafar S., Lescoat A., Huang S., Gordon J., Hinchcliff M., Shah A.A., Assassi S., Domsic R., Bernstein E.J., Steen V. (2021). Clinical characteristics, visceral involvement, and mortality in at-risk or early diffuse systemic sclerosis: A longitudinal analysis of an observational prospective multicenter US cohort. Arthritis Res. Ther..

[61] Khanna D., Lin C.J., Furst D.E., Goldin J., Kim G., Kuwana M., Allanore Y., Matucci-Cerinic M., Distler O., Shima Y. (2020). Tocilizumab in systemic sclerosis: A randomised, double-blind, placebo-controlled, phase 3 trial. Lancet Respir. Med..

[62] Roofeh D., Lin C.J., Goldin J., Kim G.H., Furst D.E., Denton C.P., Huang S., Khanna D., Investigators f. (2021). Tocilizumab prevents progression of early systemic sclerosis–associated interstitial lung disease. Arthritis Rheumatol..

[63] Khanna D., Lin C.J., Furst D.E., Wagner B., Zucchetto M., Raghu G., Martinez F.J., Goldin J., Siegel J., Denton C.P. (2022). Long-term safety and efficacy of tocilizumab in early systemic sclerosis–interstitial lung disease: Open-label extension of a phase 3 randomized controlled trial. Am. J. Respir. Crit. Care Med..

[64] Ebata S., Yoshizaki A., Oba K., Kashiwabara K., Ueda K., Uemura Y., Watadani T., Fukasawa T., Miura S., Yoshizaki-Ogawa A. (2021). Safety and efficacy of rituximab in systemic sclerosis (DESIRES): A double-blind, investigator-initiated, randomised, placebo-controlled trial. Lancet Rheumatol..

[65] Kuzumi A., Ebata S., Fukasawa T., Matsuda K.M., Kotani H., Yoshizaki-Ogawa A., Sato S., Yoshizaki A. (2023). Long-term outcomes after rituximab treatment for patients with systemic sclerosis: Follow-up of the DESIRES trial with a focus on serum immunoglobulin levels. JAMA Dermatol..

[66] Kao J.-H., Huang H.-T., Li K.-J. (2020). Nintedanib in progressive fibrosing interstitial lung diseases. New Engl. J. Med..

[67] Flaherty K.R., Wells A.U., Cottin V., Devaraj A., Walsh S.L., Inoue Y., Richeldi L., Kolb M., Tetzlaff K., Stowasser S. (2019). Nintedanib in progressive fibrosing interstitial lung diseases. New Engl. J. Med..

[68] Wells A.U., Flaherty K.R., Brown K.K., Inoue Y., Devaraj A., Richeldi L., Moua T., Crestani B., Wuyts W.A., Stowasser S. (2020). Nintedanib in patients with progressive fibrosing interstitial lung diseases—Subgroup analyses by interstitial lung disease diagnosis in the INBUILD trial: A randomised, double-blind, placebo-controlled, parallel-group trial. Lancet Respir. Med..

[69] Highland K.B., Distler O., Kuwana M., Allanore Y., Assassi S., Azuma A., Bourdin A., Denton C.P., Distler J.H.W., Hoffmann-Vold A.M. (2021). Efficacy and safety of nintedanib in patients with systemic sclerosis-associated interstitial lung disease treated with mycophenolate: A subgroup analysis of the SENSCIS trial. Lancet Respir. Med..

[70] Kolb M., Bonella F., Wollin L. (2017). Therapeutic targets in idiopathic pulmonary fibrosis. Respir. Med..

[71] Maher T.M., Corte T.J., Fischer A., Kreuter M., Lederer D.J., Molina-Molina M., Axmann J., Kirchgaessler K.U., Samara K., Gilberg F. (2020). Pirfenidone in patients with unclassifiable progressive fibrosing interstitial lung disease: A double-blind, randomised, placebo-controlled, phase 2 trial. Lancet Respir. Med..

[72] Acharya N., Sharma S.K., Mishra D., Dhooria S., Dhir V., Jain S. (2020). Efficacy and safety of pirfenidone in systemic sclerosis-related interstitial lung disease—A randomised controlled trial. Rheumatol. Int..

[73] Huapaya J.A., Silhan L., Pinal-Fernandez I., Casal-Dominguez M., Johnson C., Albayda J., Paik J., Sanyal A., Mammen A., Christopher-Stine L. (2019). Long-Term Treatment Azathioprine Mycophenolate Mofetil Myositis-RelatED Interstitial Lung Disease. Chest.

[74] Allenbach Y., Guiguet M., Rigolet A., Marie I., Hachulla E., Drouot L., Jouen F., Jacquot S., Mariampillai K., Musset L. (2015). Efficacy of rituximab in refractory inflammatory myopathies associated with anti-synthetase auto-antibodies: An open-label, phase II trial. PLoS ONE.

[75] He C., Li W., Xie Q., Yin G. (2022). Rituximab in the treatment of interstitial lung diseases related to anti-melanoma differentiation-associated gene 5 dermatomyositis: A systematic review. Front. Immunol..

[76] Hallowell R.W., Danoff S.K. (2023). Diagnosis and management of myositis-associated lung disease. Chest.

[77] Fujisawa T., Hozumi H., Kamiya Y., Kaida Y., Akamatsu T., Kusagaya H., Satake Y., Mori K., Mikamo M., Matsuda H. (2021). Prednisolone and tacrolimus versus prednisolone and cyclosporin A to treat polymyositis/dermatomyositis-associated ILD: A randomized, open-label trial. Respirology.

[78] Kameda H., Nagasawa H., Ogawa H., Sekiguchi N., Takei H., Tokuhira M., Amano K., Takeuchi T. (2005). Combination therapy with corticosteroids, cyclosporin A, and intravenous pulse cyclophosphamide for acute/subacute interstitial pneumonia in patients with dermatomyositis. J. Rheumatol..

[79] Tsuji H., Nakashima R., Hosono Y., Imura Y., Yagita M., Yoshifuji H., Hirata S., Nojima T., Sugiyama E., Hatta K. (2020). Multicenter prospective study of the efficacy and safety of combined immunosuppressive therapy with high-dose glucocorticoid, tacrolimus, and cyclophosphamide in interstitial lung diseases accompanied by anti–melanoma differentiation–associated gene 5–positive dermatomyositis. Arthritis Rheumatol..

[80] Kurasawa K., Arai S., Namiki Y., Tanaka A., Takamura Y., Owada T., Arima M., Maezawa R. (2018). Tofacitinib for refractory interstitial lung diseases in anti-melanoma differentiation-associated 5 gene antibody-positive dermatomyositis. Rheumatology.

[81] Chen Z., Wang X., Ye S. (2019). Tofacitinib in amyopathic dermatomyositis–associated interstitial lung disease. New Engl. J. Med..

[82] Wang Y., Luo J., Lv X., Li Y., An Q., Mo L., Hu N., Zhang J., Wang J., Tian J. (2023). Tofacitinib for new-onset adult patients with anti-melanoma differentiation-associated 5 gene antibody positive dermatomyositis. Clin. Rheumatol..

[83] Ida T., Furuta S., Takayama A., Tamura J., Hayashi Y., Abe K., Kurihara S., Ishikawa J., Iwamoto T., Ikeda K. (2023). Efficacy and safety of dose escalation of tofacitinib in refractory anti-MDA5 antibody-positive dermatomyositis. RMD Open.

[84] Huapaya J.A., Hallowell R., Silhan L., Pinal-Fernandez I., Casal-Dominguez M., Johnson C., Albayda J., Paik J., Lin C., Hussien A. (2019). Long-term treatment human immunoglobulin for antisynthetase syndrome-associated interstitial lung disease. Interstitial Lung Dis..

[85] Wang L.-M., Yang Q.-H., Zhang L., Liu S.-Y., Zhang P.-P., Zhang X., Liu X.-J., Han L.-S., Li T.-F. (2022). Intravenous immunoglobulin for interstitial lung diseases of anti-melanoma differentiation-associated gene 5-positive dermatomyositis. Rheumatology.

[86] Li T., Guo L., Chen Z., Gu L., Sun F., Tan X., Chen S., Wang X., Ye S. (2016). Pirfenidone in patients with rapidly progressive interstitial lung disease associated with clinically amyopathic dermatomyositis. Sci. Rep..

[87] Liang J., Cao H., Yang Y., Ke Y., Yu Y., Sun C., Yue L., Lin J. (2021). Efficacy and tolerability of nintedanib in idiopathic-inflammatory-myopathy-related interstitial lung disease: A pilot study. Front. Med..

[88] Johnson S.R., Bernstein E.J., Bolster M.B., Chung J.H., Danoff S.K., George M.D., Khanna D., Guyatt G., Mirza R.D., Aggarwal R. (2024). 2023 American College of Rheumatology (ACR)/American College of Chest Physicians (CHEST) guideline for the screening and monitoring of interstitial lung disease in people with systemic autoimmune rheumatic diseases. Arthritis Care Res..

[89] Kelly C.A., Nisar M., Arthanari S., Carty S., Woodhead F.A., Price-Forbes A., Middleton D., Dempsey O., Miller D., Basu N. (2021). Rheumatoid arthritis related interstitial lung disease–improving outcomes over 25 years: A large multicentre UK study. Rheumatology.

[90] Fernández-Díaz C., Castañeda S., Melero-González R.B., Ortiz-Sanjuán F., Juan-Mas A., Carrasco-Cubero C., Casafont-Solé I., Olivé A., Rodríguez-Muguruza S., Almodóvar-González R. (2020). Abatacept in interstitial lung disease associated with rheumatoid arthritis: National multicenter study of 263 patients. Rheumatology.

[91] Graudal N., Nielsen C.T., Lindhardsen J. (2023). Pirfenidone in rheumatoid arthritis-associated interstitial lung disease. Lancet Respir. Med..

[92] Schaefer C., Ruhrmund D., Pan L., Seiwert S., Kossen K. (2011). Antifibrotic activities of pirfenidone in animal models. Eur. Respir. Rev..

[93] Wu C., Lin H., Zhang X. (2019). Inhibitory effects of pirfenidone on fibroblast to myofibroblast transition in rheumatoid arthritis-associated interstitial lung disease via the downregulation of activating transcription factor 3 (ATF3). Int. Immunopharmacol..

[94] A Study Evaluating the Efficacy and Safety of Vixarelimab in Participants with Idiopathic Pulmonary Fibrosis and in Participants with Systemic Sclerosis-Associated Interstitial Lung Disease. https://clinicaltrials.gov/study/NCT05785624.

[95] Phase 2 Safety and Efficacy Study of Tulisokibart (MK-7240/PRA023) in Subjects with Systemic Sclerosis Associated with Interstitial Lung Disease (SSc-ILD) (MK-7240-007) (ATHENA-SSc-ILD). https://clinicaltrials.gov/study/NCT05270668.

[96] A Study of the Efficacy and Safety of Belimumab in Adults with Systemic Sclerosis Associated Interstitial Lung Disease (BLISSc-ILD). https://clinicaltrials.gov/study/NCT05878717.

[97] Determine Effectiveness of Anifrolumab In SYstemic Sclerosis (DAISY) (DAISY). https://clinicaltrials.gov/study/NCT05925803.

[98] Myositis Interstitial Lung Disease Nintedanib Trial (MINT). https://clinicaltrials.gov/study/NCT05799755.

